# Impact of phenotypic rapid diagnostic assay on duration of empiric antibiotics for gram-negative bacteremia

**DOI:** 10.1017/ash.2022.331

**Published:** 2023-01-30

**Authors:** Sana M. Mohayya, Mohammad Arsalan, Navaneeth Narayanan, Purvi Patel, Christin G. Hong, Thomas J. Kirn, Pinki J. Bhatt, Tanaya Bhowmick

**Affiliations:** 1 Robert Wood Johnson University Hospital, New Brunswick, NJ, USA; 2 Division of Allergy, Immunology, Infectious Diseases, Department of Medicine, Rutgers Robert Wood Johnson Medical School, New Brunswick, NJ, USA; 3 Rutgers University Ernest Mario School of Pharmacy, Rutgers University, New Brunswick, NJ, USA

## Abstract

**Objective:**

Rapid diagnostic tests (RDTs) are increasingly being implemented as antimicrobial stewardship tools to facilitate antibiotic modification and reduce complications related to their overutilization. We measured the clinical impact of a phenotypic RDT with antimicrobial stewardship (AMS) in the setting of gram-negative bacteremia.

**Setting and participants:**

In this single-center retrospective cohort study, we evaluated adult patients with gram-negative bacteremia who received at least 72 hours of an antibiotic.

**Methods:**

The primary outcome was the duration of empiric antibiotic therapy for gram-negative bacteremia. Secondary outcomes included time-to-directed therapy, proportion of modifications, hospital length of stay (LOS), and subsequent infection with a multidrug-resistant organism (MDRO) or *C. difficile infection* (CDI).

**Results:**

The duration of empiric antibiotics decreased in the RDT+AMS group (4 days vs 2 days; *P* < .01). Time to directed therapy decreased from 75.0 to 27.9 hours (*P* < .01).

**Conclusions:**

The clinical outcomes of LOS, MDRO, and CDI were reduced. The phenotypic RDT demonstrated an improvement in stewardship measures and clinical outcomes.

Bloodstream infections (BSIs) are a common cause of morbidity and mortality worldwide, of which gram-negative bacteria account for 25%–50%.^1,2^ Morbidity and mortality are significantly higher in patients who do not receive appropriate antibiotic therapy in a timely manner.^3^ Empiric use of broad-spectrum antibiotics for extended periods is a common misuse of antibiotics, which can result in the emergence of resistant pathogens, an increase in *C. difficile* (CDI) occurrence, prolonged hospital stays, and a higher mortality rate.^4–6^

In the United States, the determination of the etiology of BSI begins with the detection of bacteria in blood specimens incubated in an automated blood-culture system. Bacterial identification and antimicrobial susceptibility testing are performed after growth on agar plates to yield isolated bacterial colonies. The entire process can take up to 2–3 days.^7^ During this time, patients are receiving empiric antimicrobial therapy that may be either suboptimal or of unnecessarily broad spectrum. Rapid diagnostic tests (RDTs) are being increasingly implemented with the goal of improving antibiotic use and reducing the negative sequelae of treatment. Studies have illustrated the potential efficacy of RDTs, including the theoretical benefit of a phenotypic RDT, which may be associated with more rapid de-escalation and a reduction in broad-spectrum antibiotic use.^8–10^ Subsequent studies have substantiated that this RDT may improve the time to directed therapy and duration of therapy.^11–13^ However, despite the improvement in these measures, some studies did not associate them with improved clinical outcomes. For example, one study demonstrated similar improvements in stewardship outcomes, but no significant improvement in clinical outcomes such as length of stay (LOS), CDI or mortality.^14^ Another study reported similar results but also illustrated the potential for erroneous results, suggesting that other antimicrobial stewardship (AMS) measures must be used concomitantly.^15^

The phenotypic RDT implemented at our institution is the Accelerate PhenoTest (Accelerate Diagnostics, Tucxon, AZ), a rapid phenotypic diagnostic instrument used to identify microorganisms in blood samples in <2 hours and provides antimicrobial susceptibility results in ∼7 hours. This system was designed to expedite the identification and susceptibility testing of certain microorganisms in the blood compared to a culture-based system.^16^ Accelerate PhenoTest can identify several fungal pathogens, as well as gram-positive and gram-negative bacteria. Specifically, our institution implemented this instrument to identify only gram-negative pathogens in blood cultures. The gram-negative bacteria that can be identified through the test include *Acinetobacter baumannii*, *Citrobacter* spp, *Enterobacter* spp, *Escherichia coli*, *Klebsiella* spp, *Proteus* spp, *Pseudomonas aeruginosa*, and *Serratia marcescens*.

In this study, we measured the clinical impact of a phenotypic RDT compared to standard laboratory practice in the setting of gram-negative bacteremia. We hypothesized that the implementation of this RDT with AMS measures would be associated with shorter durations of empiric antibiotics and improved clinical outcomes.

## Methods

### Study design

This single-center, retrospective, observational, before-and-after cohort study was conducted at a 625-bed academic medical center. Data were collected via electronic medical record (EMR) chart reviews of inpatient encounters from June 2018 to November 2018 (historical cohort) and from June 2019 to November 2019 (RDT cohort). Convenience sampling of the first episode per patient was conducted during the specified study period. The EMR used at the institution was Sunrise Clinical Manager (Allscripts, Chicago, IL). Our study was approved by the Rutgers Institutional Review Board and was performed in accordance with the ethical standards laid down in the 1964 Declaration of Helsinki and its later amendments. Our study did not include factors that required patient consent.

### Participants

We included hospitalized adults (≥18 years) with gram-negative bacteremia, as indicated by a positive blood culture for a gram-negative pathogen, who received at least 72 hours of systemic antibiotics. A period of 72 hours was chosen because final culture susceptibilities are usually reported within that period. Patients were excluded if they were transferred from an outside facility because the choice of antibiotics would be based on culture results from the outside facility, resulting in inaccurate timing of antibiotics. Polymicrobial infections, defined as >1 pathogen identified in 1 set of blood cultures, were also excluded because other culture results may influence the provider’s decision to continue or modify antimicrobial therapy. Patients who died or were discharged prior to culture results were also excluded.

### Reporting from the microbiology laboratory

The microbiology laboratory is located at Robert Wood Johnson University Hospital. The blood-culture sets consisted of 1 BD BACTEC Plus and 1 BD BACTEC Lytic blood culture bottle (Becton Dickinson, Franklin Lakes, NJ), each ideally filled with 8–10 mL blood. Incubation was performed using the BACTEC FX instrument and Gram-stain results from all single positive blood cultures were called to the nurse caring for the patient, who then informed the patient’s physician. Final culture results with identification of organism and susceptibility to antimicrobials are available in the EMR. This system of reporting occurred during both the historical and intervention periods.

### Microbiology procedure

*Historical period.* Upon identification of a gram-negative organism on the Gram stain of a signal positive blood-culture bottle, specimens were plated on blood, chocolate, and MacConkey agar plates. Approximately 24 hours later, bacterial colonies were identified using the Bruker MALDI-TOF instrument (Bruker, Billerica, MA) and antimicrobial susceptibility testing (AST) was performed using the BD Phoenix automated identification and susceptibility testing system (Becton Dickinson). Final identification and AST results were reported in the EMR ∼48–72 hours after blood culture bottles signaled positive for growth. No antimicrobial stewardship interventions for gram-negative bacteremia were in place during this period.

*Intervention period.* In May 2019, the microbiology laboratory implemented the Accelerate PhenoTest system (Tucson, AZ), which provides identification and susceptibility results from blood cultures for a limited number of microorganisms within ∼8 hours. The baseline procedures were continued as noted. The results were reported in the EMR. Updated results with additional antibiotic susceptibility results from the Phoenix were entered into the EMR when they became available. If there was a discrepancy between the results of the 2 instruments, additional testing was performed at the discretion of the microbiology laboratory directors and the antimicrobial stewardship response team (ASRT) notified the primary team of the final results. In addition, the microbiology technologists emailed the organisms’ identifications and susceptibility results to the ASRT via electronic communication 24 hours a day and 7 days a week. A phone call was also placed to the ASRT during business hours on weekdays (excluding holidays) and at all other times to the nurse caring for the patient. The ASRT consisted of infectious diseases (ID) faculty members, including physicians and an ID pharmacist, who were responsible for reviewing all gram-negative culture results from the Accelerate PhenoTest and making recommendations to the primary team responsible for the patient. The ASRT also reviewed other RDT results that were not bacteria related and would not affect the patient population studied. If results were reported outside regular business hours, they were e-mailed to the ASRT, who would communicate the results and recommendations the following day. During the RDT period, the microbiology laboratory added personnel to the evening shift, so results were reported during evening hours. Educational efforts were made to all ordering providers prior to the implementation via in-person sessions and electronic distribution through the medical staff newsletter. We implemented RDT and ASRT simultaneously based on experiences at our institution that demonstrated physicians responded to direct prompts to act on results in a timely manner.^17^

### Outcomes

The primary outcome was the total duration of empiric antibiotic therapy for gram-negative bacteremia from the time of the Gram stain. Secondary outcomes included modification of therapy within 24 hours of the reported susceptibilities. Modification was defined as either a de-escalation to a targeted agent or escalation of therapy to broader coverage based on the RDT results. De-escalation was defined as a change in the antibiotic from a broad-spectrum agent to a more targeted agent based on the RDT results. Escalation was defined as a change in the antibiotic to one with a broader spectrum based on the RDT results. Broad-spectrum antibiotics consisted of, but were not limited to, piperacillin-tazobactam, cefepime, meropenem, aztreonam, and ceftazidime, whereas narrow-spectrum antibiotics included, but were not limited to, ceftriaxone, cefazolin, ampicillin, and levofloxacin. Notably, many patients were not started on broad-spectrum antibiotics, and for many patients, targeted therapy did not include a narrow-spectrum antibiotic. All outcomes measuring time were measured from the time of Gram stain (“time zero”). We chose Gram stain rather than blood-culture draw to ensure accurate time measurements for antibiotic changes based on results reported by the microbiology laboratory and to appropriately assess the impact of the RDT. Antibiotics were not assessed prior to Gram stain results. Other outcomes included time to directed therapy, modification within 24 hours of susceptibility results, modification at any point in therapy, modified antibiotic from empiric choice if the antibiotic was modified from empiric therapy, hospital LOS, 14-day in-hospital mortality, subsequent infection with a multidrug-resistant organism (MDRO) within 30 days of the initial positive culture, and development of CDI within 30 days of initial positive culture. Time to directed therapy was defined as the time from the Gram stain result report to the time of escalation or de-escalation to an antibiotic with in vitro activity against the pathogen, and only included patients who had a modification of therapy. *Clostridioides difficile* testing was performed using a 2-step algorithm using GDH-toxin A/B EIA with reflex to PCR if GDH positive and toxin negative. The process was the same during both the historical and intervention periods. MDRO was defined as a pathogen that had resistance to ≥3 classes of antibiotics.

### Statistical analysis

Continuous data are reported as means with standard deviations or medians with interquartile ranges (IQR), as appropriate. All categorical data are reported as percentages. Statistical significance for continuous data was determined using the Student *t* test for parametric variables or the Wilcoxon rank-sum test for nonparametric variables. Categorical data were analyzed using the χ^2^ or the Fisher exact test, as appropriate. The significance level was determined as a 2-sided *P* value of <.05. A subgroup analysis of the baseline characteristics was performed for patients who did not achieve the primary outcome. Data were analyzed using R software version 1.4.11.06 software (R Foundation for Statistical Computing, Vienna, Austria).

## Results

**Fig. 1.**
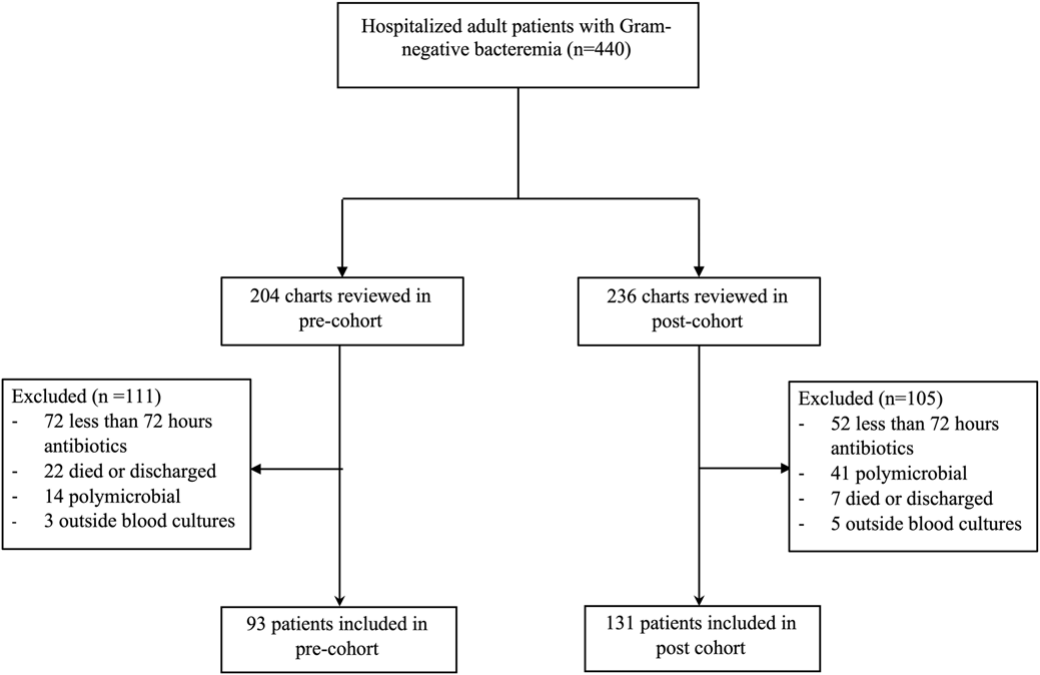
Study flow chart of included study participants.

In total, 440 charts were reviewed, of which 93 in the preintervention cohort and 131 in the postintervention cohort met inclusion criteria (Fig. 1). Patients were well matched based on basic demographics and severity of illness scores. The most commonly isolated pathogen was *E. coli*, followed by *Klebsiella pneumonia* (Table 1). No patients in the preintervention cohort had *Pseudomonas*. The timing of result reporting differed between the 2 groups, 98% and 63% were reported during business hours, respectively (*P* < .01).

**Table 1.** Baseline Demographics and Clinical Characteristics of Patients Included in the Study

Characteristic	Preintervention Cohort (n=93), No. (%)^a^	Postintervention Cohort (n=131), No. (%)^a^	*P* Value
**Patient demographics**			
Age, median y (IQR)	65 (58–77)	66 (57–77)	.80
Sex, male	45 (48)	71 (54)	.47
Chronic kidney disease	18 (19)	27 (21)	.95
**Encounter characteristics**			
Pitt bacteremia score, median (IQR)	2 (1–3)	1 (0–2)	.10
Charlson comorbidity index (CCI), median (IQR)	4 (3–6)	5 (3–7)	.35
Pregnant	0 (0)	1(0.01)	1.0
ICU admission while on antibiotic	37 (40)	48 (37)	.74
ICU length of stay, median d (IQR)	5 (3–12)	5 (2–13)	.68
**Isolated taxon**			.13
*Escherichia coli*	49 (53)	71 (54)	
*Klebsiella* spp	26 (28)	24 (18)	
*Enterobacter* spp	9 (10)	14 (11)	
*Proteus* spp	4 (4)	12 (9)	
*Serratia marcescens*	2 (2)	3 (2)	
*Pseudomonas aeruginosa*	0 (0)	6 (5)	
*Acinetobacter baumannii*	1 (1)	1 (0.7)	
*Stenotrophomonas maltophilia*	2 (2)	0 (0)	
**Resistance^b^**			
Carbapenems	5 (5)	4 (3)	.60
Third generation cephalosporins	13 (14)	29 (22)	.17
Fluoroquinolones	20 (22)	37 (28)	.33
Empiric vancomycin therapy	30 (32)	27 (21)	.07
Allergies	23 (25)	27 (21)	.57
β-lactam allergy	11 (12)	13 (10)	
Antibiotic allergy in addition to β-lactam	5 (5)	5 (4)	
Other antibiotic allergy (non–β-lactam)	7 (8)	9 (7)	
Neutropenic	14 (15)	16 (12)	.68
**Likely source of bacteremia, if known^c^**			0.15
Urinary only	38 (41)	64 (49)	
Abdominal only	22 (24)	26 (20)	
Respiratory only	6 (6)	4 (3)	
Skin and soft-tissue only	1 (1)	3 (2)	
Unknown	7 (8)	19 (15)	
Other^d^	19 (20)	15 (11)	
Other positive culture^e^	38 (41)	67 (51)	0.17
Same pathogen as index blood culture^f^	34 (89)	58 (87)	0.77
Same sensitivity profile as index blood culture^g^	31 (91)	56 (97)	0.53
Removed focus of infection^h^	30 (83)	19 (86)	0.99
Susceptibilities reported during day^i^	91 (98)	82 (63)	<0.01
Infectious disease consultation	72 (77)	98 (75)	0.85
**Antibiotic at time susceptibilities reported**			0.88
Piperacillin-tazobactam	51 (55)	77 (59)	
Ceftriaxone	9 (10)	11 (8)	
Meropenem	15 (16)	17 (13)	
Aztreonam	4 (4)	5 (4)	
Cefepime	7 (8)	15 (11)	
Ceftazidime	2 (2)	2 (2)	
Other^j^	5 (5)	4 (3)	
**Primary admitting team**			0.15
Internal medicine	44 (47)	84 (64)	
Intensive care	24 (26)	22 (17)	
Hematology/Oncology	10 (11)	9 (7)	
Surgical	7 (8)	9 (7)	
Other^k^	8 (9)	7 (5)	
**Discharge status**			0.86
Home	48 (52)	69 (53)	
Institution^l^	36 (39)	52 (40)	
Death^m^	9 (10)	10 (8)	

Note. CKD, chronic kidney disease; ICU, intensive care unit; ID, infectious disease.

a Data are presented as no. (%) unless otherwise indicated.

b Proportion of isolates not susceptible to one or more antibiotic in each class. Carbapenem and third-generation cephalosporin categories are mutually exclusive; isolates not susceptible to carbapenems are not included in the third-generation cephalosporin category.

c Source of bacteremia is reported as the proportion of patients who had an identified source of infection. The source was identified in 94 patients in the preintervention cohort and 96 patients in the postintervention cohort.

d Other sources of infection included surgical site, cardiac, and multisite infections.

e A positive culture from another source (ie, urine, tissue, body fluid) which had final results prior to or within 24 h of final blood-culture results.

f Proportion of other cultures with same pathogen are reported based on the frequencies of patients with other positive cultures.

g Proportion of other cultures with same sensitivities are reported based on the frequencies of cultures with same pathogen as blood culture.

h Removed focus of infection includes patients who had a source of infection which may require removal to achieve source control. The percentages reported are based on the total number of patients who had a removable source of infection. This included 36 patients in the preintervention cohort and 22 patients in the postintervention cohort.

i Time of when susceptibilities were called to the respective provider. If a call was performed during normal business hours, or 9:00 a.m. to 5:00 p.m., this was considered day shift.

j Other antibiotics include: aztreonam, levofloxacin, ertapenem, ampicillin-sulbactam, ciprofloxacin, ceftazidime-avibactam, ceftolozane-tazobactam.

k Other services include gynecology/oncology, cardiology, neurology, nephrology, urology.

l Institution is defined as discharged to another institution, including another hospital, rehabilitation center, long-term care facility, or skilled nursing facility.

m Death includes patients who were discharged to hospice.

The primary outcome of duration of empiric antibiotic therapy decreased from 4 days to 2 days (*P* < .01) in the RDT+AMS group (Table 2). Most patients were treated with empiric piperacillin-tazobactam or meropenem and were transitioned to ceftriaxone or levofloxacin. The time to directed therapy decreased from 75.0 hours to 27.9 hours (*P* < .01). Modification within 24 hours of susceptibility results was also more common in the RDT+AMS group (66% vs 47%; *P* = .02) among patients who had a modification of therapy. Among all patients, the modification within 24 hours of susceptibility results was 32% in the historical group and 56% in the RDT group (*P* < .01).

**Table 2.** Primary and Secondary Outcomes

Outcome	Preintervention Cohort (n=93), No. (%)^a^	Postintervention Cohort (n=131), No. (%)^a^	*P* Value
**Primary outcome**			
Duration of empiric antibiotics, median d (IQR)	4.0 (3.6–5.9)	2.0 (1.6–3.2)	<.01
**Secondary outcomes**			
Modification within 24 h of susceptibility results^b^	30 (47)	73 (66)	.02
Modification within 24 h of susceptibility results among all patients^c^	30 (32)	73 (56)	<.01
Modification at any point in therapy	64 (69)	111 (85)	.01
Modified antibiotic^d^			<.01
Ceftriaxone	14 (22)	55 (50)	
Levofloxacin	16 (25)	7 (6)	
Cefazolin	10 (16)	9 (8)	
Meropenem	5 (8)	11 (10)	
Piperacillin-tazobactam	0 (0)	5 (5)	
Cefepime	0 (0)	3 (3)	
Other^e^	19 (30)	21 (19)	
Time to directed therapy from Gram stain, median h (IQR)^f^	75.0 (68.7–96.5)	27.9 (18.6–44.7)	<.01
Time to directed therapy from susceptibility results, median h (IQR)^g^	25.8 (9.4–51.0)	18.9 (8.7–28.1)	<.01
Days of therapy of empiric antibiotics, median d (IQR)	5 (4–6)	3 (2–4)	<.01
14-day in-hospital mortality	6 (6)	6 (5)	.57
Length of stay, median d (IQR)	12.2 (6.8–23.4)	9.0 (6.0–20.5)	.09
Subsequent MDROs within 30 d	10 (11)	2 (2)	.01
*C. difficile* infection within 30 d	8 (9)	1 (0.9)	.01

Note. AKI, acute kidney injury; DOT, days of therapy.

a Data are presented as no. (%) unless otherwise indicated.

b If antibiotic was modified. Percentages are calculated based on the total number of patients who modified therapy. In the pre-intervention cohort, 64 patients had a modification of therapy, and in the post-intervention cohort 111 patients had a modification.

c Percentages are calculated based on the total number of patients who modified therapy among all patients included in the study.

d Modified antibiotic chosen after change from empiric choice. Percentages are calculated based on the total number of patients who modified therapy.

e Other antibiotics after modification include amoxicillin, amoxicillin-clavulanate, ampicillin-sulbactam, sulfamethoxazole-trimethoprim, ceftazidime, ceftazidime-avibactam, ceftolozane-tazobactam, cephalexin, ciprofloxacin, ertapenem, meropenem-vaborbactam. In cases when escalation of therapy was indicated, piperacillin-tazobactam and meropenem were initiated.

f Time to modification among patients who modified at any point in therapy. This was calculated from the time of the Gram stain result report to the time the directed antibiotic was ordered.

g Time to modification among patients who modified at any point in therapy. This was calculated from the time of the susceptibility results to the time the directed antibiotic was ordered.

The total hospital LOS decreased from 12.2 days in the RDT+AMS group to 9.0 days in the historical group, but this difference was not statistically significant (*P* = .09). The subsequent development of MDRO within 30 days was lower in the RDT+AMS group, with 11% in the control group and 2% in the RDT+AMS group (*P* = .01). A similar difference was observed with CDI, with a difference of 9% in the control group compared with 0.9% in the RDT+AMS group (*P* = .01).

## Discussion

We evaluated the effect of a phenotypic RDT on antibiotic usage for gram-negative bacteremia when used with active antimicrobial stewardship practices. The primary outcome of the duration of empiric antibiotics was significantly reduced, suggesting a positive effect of the RDT+AMS combination. The secondary outcomes of modification at any point in therapy, time to directed therapy among patients who had a modification in therapy, MDROs and CDI were statistically significant. The LOS decreased, although this was not statistically significant. There were no differences in in-hospital mortality. Most patients were modified from a broad-spectrum β-lactam, most commonly piperacillin-tazobactam, to a narrower-spectrum β-lactam, most frequently ceftriaxone.

The reduction in empiric antibiotic days is an important finding, as each day of inappropriate antibiotic therapy is associated with a higher likelihood of negative outcomes.^18^ This finding was further demonstrated by a reduction in subsequent MDRO and CDI. These improved outcomes may have led to a reduction in the median LOS. Although this was not statistically significant, the trend is noteworthy and may suggest that the RDT may be an important cost savings measure when used with active AMS strategies.

The proportion of patients who had a modification of therapy within 24 hours of susceptibility results was high in both the pre- and postintervention periods. Although the RDT would have affected the time to the result, the time to modification after the result was reported would be less likely to be affected by the instrument alone. However, the collaboration with the ASRT, in which physicians were contacted with real-time results during daytime hours, should have improved this. In addition, the time to directed therapy from Gram stain was significantly reduced in the postintervention period. More importantly, modifications at any point in therapy occurred more often in the postintervention period. This may have been due to the faster results and/or ASRT involvement with patient care, both of which would allow providers more time to assess the patient’s clinical status and other factors, and to make a confident and timely decision.

Our findings are generally consistent with those of previously published studies assessing the impact of a phenotypic RDT. These studies demonstrated an improvement in antimicrobial stewardship measures, primarily in the time to directed therapy. Banerjee et al^19^ found a faster median time to first antibiotic modification, and MacVane et al^14^ concluded that RDT had an improved the time to optimal therapy, both of which are consistent with our findings. Babowitz et al^11^ analyzed the RDT and found improved utilization of gram-negative antibiotics in patients with sepsis. Our study adds to the literature by broadening these findings to all patients with gram-negative bacteremia, not just critically ill patients, suggesting a greater real-world impact. Furthermore, our study period was conducted prior to the COVID-19 pandemic, so we did not include patients with concurrent COVID-19, which may have affected others’ results.^11^

Despite the clear improvements in antimicrobial usage, the published data reports conflicting results regarding the impact of the RDT on clinical outcomes. Although some studies did not find any significant improvements in clinical outcomes, others did.^11–13,15,19^ Dare et al^13^ and Walsh et al^12^ found an improvement in the LOS, similar to our results. Babowicz et al^11^ suggested a potential improvement in mortality. Although our results did not show an improvement in inpatient mortality, the reduction in days of empiric antibiotics may potentially improve long-term outcomes. Teshome et al^18^ concluded that each additional day of unnecessary antipseudomonal β-lactam therapy may increase the risk of further resistance. This was illustrated by our decrease in the number of subsequent MDRO infections.

The limitations of our study include its retrospective design, which may not account for all confounding factors. However, careful consideration was taken to limit the confounding factors. By including only gram-negative bacteremia patients, we believe that we have mitigated the risk of including patients with contaminated blood cultures that may not require treatment. Another consideration is that the timing of the reporting of susceptibilities had changed in the postintervention cohort owing to scheduling changes. In the control group, most results were reported during business hours (9:00 a.m. to 5:00 p.m.), while the RDT+AMS group had the results reported during both the day and evening shifts, with significantly fewer reported during the day shift. Most therapy changes occur during the day shift at our institution, so the decrease in reporting during business hours would result in slower modifications in the intervention group because results reported late would be reviewed the next business day. Notably, time to directed therapy from susceptibility results supports that the change in reporting times did not affect our results. This finding may further solidify the positive impact of the RDT. Additionally, it is evident that the stewardship component was effective because the modification in therapy within 24 hours of the susceptibility results was statistically significant. We did not perform an analysis regarding acceptance or rejection of ASRT recommendations to correlate its impact; however, we were able to demonstrate an impact of the combined intervention.

The greatest strength of our study is its real-world impact assessment. Our primary outcome of the duration of empiric antibiotics differs from that of other studies that investigated the time to directed therapy. The latter would have excluded patients who did not have a modification of therapy or who did not achieve directed therapy during the hospital course. By including all eligible patients, our data would be a better representation of the true effect of the intervention. Ours is the first study to show an improvement in MDRO and CDI. Despite the low incidence of each infection, we were still able to detect a difference. However, further studies directly examining the effect of RDT and stewardship measures on the incidences of CDI and MDRO are needed to draw definitive conclusions.

These data support the use of a phenotypic RDT in combination with an active AMS intervention. The adoption of this instrument with AMS tools may improve antimicrobial usage, which in turn may improve the associated clinical outcomes.

In conclusion, the results of our study demonstrate that a rapid phenotypic diagnostic test positively influences antibiotic usage for gram-negative bacteremia. The duration of empiric antibiotics and time to directed therapy decreased, but modifications of therapy increased. Clinical outcomes of subsequent MDROs and CDI improved with the RDT+AMS. Further studies are required to investigate the clinical significance of these discrepancies.
